# Expression and distribution of dendritic cells in nasal polyps

**DOI:** 10.3892/etm.2013.986

**Published:** 2013-03-01

**Authors:** XIN-SHENG LIN, XIAN-YANG LUO, HUI-GE WANG, CHUANG-WEI LI, XIN LIN, CHU YAN

**Affiliations:** 1 Department of Otolarynology-Head and Neck Surgery, The Shantou Central Hospital/Affiliated Shantou Hospital of Sun Yet-Sen University, Shantou, Guangdong 515041;; 2 Department of Otorhinolaryngology Head and Neck Surgery, The First Affiliated Hospital of XiaMen University, Xiamen, Guangdong 361003;; 3 Department of Otolarynology-Head and Neck Surgery, The First Affiliated Hospital of Medical College of Shantou University, Shantou, Guangdong 515041, P.R. China

**Keywords:** nasal polyps, dendritic cells, S-100 protein, CD1a, CD40, pathogenesis

## Abstract

The aim of the present study was to investigate the expression, distribution and function of dendritic cells (DCs) and to study their role in nasal polyps. The study involved 55 participants, 45 of whom had nasal polyps and were the study group and 10 who had normal inferior turbinates and were the control group. Immunohistochemical staining was used to visualize the expression and distribution of the S-100 protein. A double immunostaining method was used to visualize the CD1a and CD40 expression and the images were analyzed with Axioplan 2 microscopy. The expression level of the S-100 protein in the nasal polyps was higher than that in the normal inferior turbinates with a significant difference (P<0.01). The distribution area, number and density of the double stained cells in the nasal polyps were all greater than in the normal inferior turbinates (P<0.01). The S-100 protein and double stained cells were mainly located in the lamina propria below the mucous membrane. The present study demonstrates that DCs are involved in the pathogenesis of nasal polyps and the presence of CD40-positive DCs suggests that this was related to the reciprocal interaction between the DCs and T lymphocytes.

## Introduction

Dendritic cells (DCs) are the most potent antigen-presenting cells (APCs) and are able to induce primary immune responses. DCs possess the capability to capture, process and present antigens and induce immune diseases. DCs are special APCs which are able to present antigens to naïve and quiescent T cells and consequently play a significant role not only in the initiation but also in the maintenance of inflammation and allergic diseases (1). Nasal polyps are frequently presented at the Department of Otolarynology-Head and Neck Surgery, The Shantou Central Hospital/Affiliated Shantou Hospital of Sun Yet-Sen University, Shantou, China. The pathogenesis of nasal polyps is not clear and now the majority of scholars believe that nasal polyps are the result of a number of complications and that the pathological immune system may play a key role in their onset and development. The penetration of T lymphocytes and eosinophils (EOSs), an imbalance in the proportion of T helper 1 to T helper 2 (Th1/Th2) cells and a predominance of the Th2 cell cytokines are characteristics of nasal polyps. DCs are able to present antigens to T cells and activate them through co-stimulating molecules and cytokines. Therefore DCs are likely play a significant role in the pathogenesis of nasal polyps. However, there have not been any direct studies into the correlation between DCs and nasal polyps. Therefore, the present study aimed to investigate the expression, distribution and function of DCs in nasal polyps and also to study their role in nasal polyp pathogenesis. It is known that the S-100 protein is a non-specific marker of DCs and that CD1a/CD40 double immunostaining is a comparatively specific marker of DCs. CD40 is a significant co-stimulating molecule that causes DCs to activate T cells. In the present study, immunohistochemical and double immunostaining methods were used to detect the expression and distribution of S-100 and CD1a/CD40 in the nasal polyps, to gain information concerning the role of DCs in their pathogenesis.

## Subjects and methods

### Subjects

The study group consisted of 45 patients with nasal polyps (25 males and 20 females), ranging in age from 11–74 years (mean, 35.53 years) and 10 patients (8 males and 2 females), 17–62 years old, (mean, 34 years) with a deviation of the nasal septum requiring surgical treatment that were assigned as the control. The cases were diagnosed by histological examination (hematoxylin and eosin staining). None of the patients received any type of corticosteroid therapy for 30 days prior to surgery. The study was conducted in accordance with the Declaration of Helsinki and with approval from the Ethics Committee of Shantou Central Hospital. Written informed consent was obtained from all patients and control subjects after the nature and purpose of the study had been explained. The nasal polyp and normal inferior turbinate tissue specimens were collected during surgery. All samples were fixed in 10% buffered formalin, embedded in paraffin and cut into 4-*μ*m sections.

### Staining methods

An immunohistochemical staining Elivision™ two-step method was performed to detect the S-100 protein. The kits were provided by Fuzhou Maixin Biotechnology Development Co., Ltd., Fuzhou, China. A rabbit anti-human polyclonal antibody to the S-100 protein (Pharmingen International, San Diego, CA, USA) was used as the primary antibody and a mouse anti-rabbit IgG monoclonal antibody was used as the secondary antibody. 3,3′-Diaminobenzidine (DAB)-hydrogen peroxide was used as a chromogen.

The staining of CD1a and CD40 was performed with certain modifications according to the previous S-100 staining method. Two types of primary antibody [mouse anti-human monoclonal antibody to CD1a (Lab Vision Corporation, Waltham, MA, USA) and rabbit anti-human monoclonal antibody to CD40 (Santa Cruz Biotechnology, Inc., Santa Cruz, CA, USA)] were added to the specimens at the same time. The specimens were incubated at 40°C overnight and then washed with PBS three times. The secondary antibodies (anti-mouse antibody-FITC, KPL; anti-rabbit antibody-PE, Santa Cruz Biotechnology, Inc.) were added and the specimens were rein-cubated at 37°C with the avoidance of light for 1.5 h.

A known esophagal carcinoma sample was used as a positive control; the negative control slides were processed with PBS liquid instead of the primary antibody, but included all other steps of the procedure.

Expression of the S-100 protein was located in the nucleus and cytoplasm of the DCs. Expression was determined using Frowits’s method with double-blind reading by two independent observers. Five fields of view in 400 were chosen for determination. Appearance of earthy yellow or brown-yellow granules in the cytoplasm was considered as a positive result. The percentage of positive cells and staining intensity in five fields of view were calculated and analyzed, and the cell grading was performed as follows: i) According to the percentage of positive cells, grade 0: no cell coloration or positive cell percentage <5%; grade 1: 5–35% of cells were colored; grade 2: 36–65% of cells were colored; grade 3: more than 65% of cells were colored. ii) According to color depth, grade 0: no cell coloration or unclear coloration; grade 1: earthy yellow; grade 2: brown-yellow; grade 3: seal brown. The final score was the average value of above two grading methods. Scores of 0, 0.5–1 and 1.5–3 were defined as negative (−), positive (+) and strong positive (++), respectively. The positive staining intensity of S-100 was calculated using an Image-pro plus 5.0 analysis system (Media Cybernetics, Inc., MD, USA) and presented as the IOD (integrated optical density, IOD = average positive grey value-average positive background value).

CD1a and CD40 were expressed in the cytoplasm of the DCs. Positive staining of CD1a was demonstrated by olivine fluorescence and that of CD40 by a red fluorescence. The double immunostaining of CD1a and CD40 produced a yellow fluorescence. Every slice was taken five eye-shot in 400 times and analyzed using a KS400 analysis system (Carl Zeiss, Inc., New York, NY, USA) to check the distribution areas, quantity and density of the DCs.

### Statistical analysis

Statistical calculations were performed using SPSS 10.0 (SPSS, Inc., Chicago, IL, USA) statistical software. The data are expressed as mean ± SD. The unpaired and paired t-test was used to compare the staining intensities of S-100, the distribution areas and the quantity and density of the double immunostain-positive cells. The S-100 and CD1a/CD40-positive rates were shown as percentages and the data were analyzed by the χ^2^ test. P<0.05 was considered to indicate a statistically significant difference.

## Results

### Expression of S-100 protein

**Figure 1:**
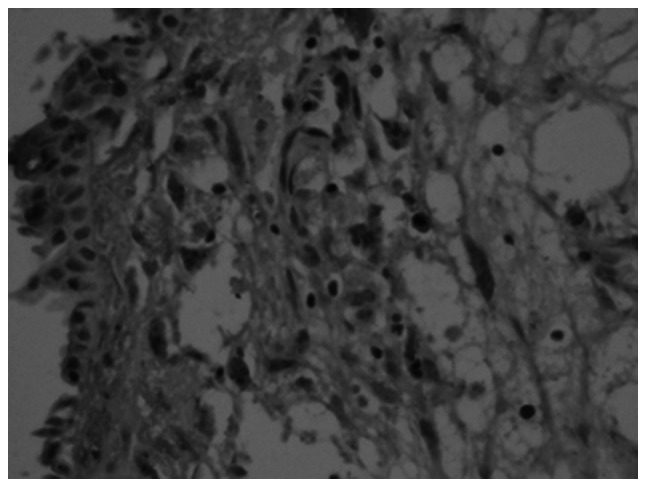
Expression of S-100 protein in nasal polyp tissues (3,3′-diaminobenzidine, magnification, ×400).

The S-100 protein-positive DCs were mainly distributed in the submucosa of the nasal polyps (Fig. 1). In the inferior turbinate tissues lined with ciliated epithelium, there were almost no S-100 protein-positive cells. The DCs were S-100 protein-positive in the nasal polyp tissues in 40 cases; the positive rate was 88.9%. Correspondingly, only one case from the control group was weakly positive for the S-100 protein. When analyzed by IOD, the inferior turbinate tissues were shown to seldomly express S-100 protein (with a staining intensity of 890.02±723.24). By contrast, the nasal polyp tissues had elevated expression levels of the S-100 protein (with a staining intensity of 8540.12±1249.79; Table I).

### Expression of CD1a/CD40

**Figure 2:**
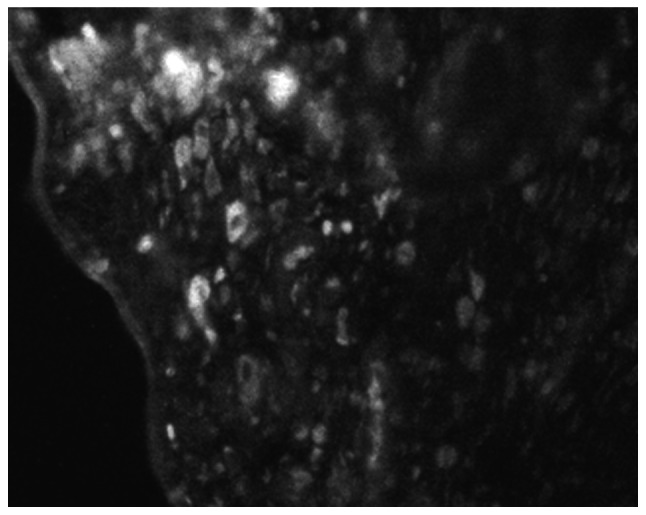
Cells double stained for CD1a and CD40 in nasal polyp tissues (magnification, ×400).

**Figure 3:**
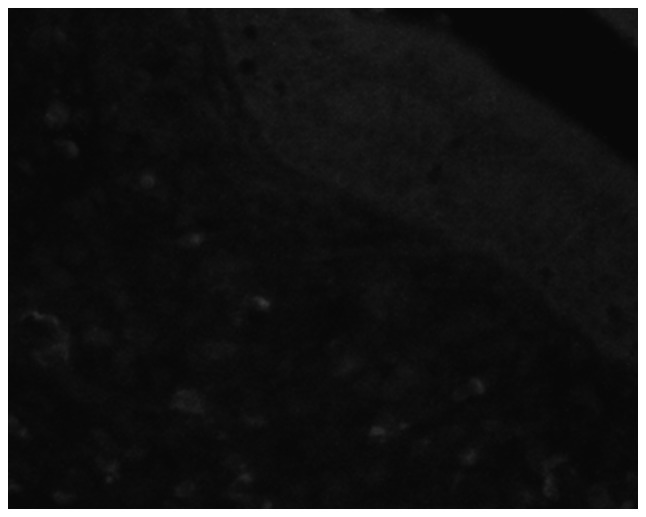
Rare cells double stained for CD1a and CD40 in normal inferior turbinate (magnification, ×400).

As was observed for S-100, the CD1a and CD40 double stained-positive cells were mostly in the submucosa of the nasal polyps (Fig. 2). In the inferior turbinate tissues, there were rarely any double stained-positive cells (Fig. 3). The positive rates were 88.9 and 10.0% in the nasal polyps and inferior turbinate tissues, respectively. The difference in the area, number and density of the double stained cells between the control and study groups was statistically significant (P<0.01; Table II).

## Discussion

DCs are the most important APCs and are extremely rare in normal tissues. Aimed at a variety of antigens, DCs express various types of TOLL-like receptors and decide the type of immunoreation (2). DCs are able to capture antigens at the lymphocyte, process them into antigenic epitopes and link with MHC II. DCs are activated following the presentation of the MHC II-antigenic epitopes to the T cells and produce numerous types of cytokines and chemokines. The chemokines may then collect more DCs that are assembled in the pathological tissues. DCs and T cells interact with each other through co-stimulating molecules and cytokines. The DCs process the antigenic epitope to the T cells and upregulate the CD40L on their surface. The CD40L connects with the CD40 of the DCs to activate the B7 molecules (CD80/CD86). The B7 molecules of the DCs activate the CD28/CTLA-4 of the T cells, inducing the activation of the cells and resulting in an immunoreaction (3).

The pathogenesis and mechanisms behind nasal polyps are unclear. Bernstein’s hypothesis (4) has been accepted by the majority of scholars. The effects of the changes in nasal aerodynamics, inflammation and allergic factors induce serious inflammation in the nasal mucosa. The infiltration of large numbers of inflammatory cells and the effects of inflammation mediators lead to the nasal mucosa becoming edemic. This causes a breaks in the epithelium, submucosal extrusion and epithelialization, thus creating nasal polyps.

The causes of nasal polyps include allergies, genetic predisposition, autonomic dysfunction of the blood vessels of the nasal mucosa and inflammation (4). The infiltration of inflammatory cells, particularly eosinophils (EOSs) and T lymphocytes, and abnormalities in the cytokines, including the upregulation of interleukin (IL)-4, IL-5 and IL-10, are characteristics of the immune dysfunction of nasal polyps (5,6). The increase in the number of T lymphocytes, the imbalance in the proportion of Th1/Th2 cells and the predominance of cytokines produced by the Th2 cells have been proposed to be the most significant immunological abnormalities (7).

The present study demonstrated that the presence of DCs (S-100 protein-positive cells) in the normal inferior turbinate tissue was rare. Compared with in the turbinates, the number of DCs in the nasal polyps was markedly increased. This suggested that DCs may play a significant role in the pathogenesis of nasal polyps.

In addition, the CD40-positive nature of the surface of the DCs suggested that DCs may react in the nasal polyps through reciprocal interaction with T lymphocytes. The S-100 protein and double stained cells were mainly located in the lamina propria below the mucous membrane. Yoshimi *et al*(8) reported that DCs were only detectable in the squamous epithelium and the authors believed that the migration of DCs into the squamous epithelium may have been regulated by cytokines, including IL-1 and granulocyte-macrophage colony-stimulating factor (GM-CSF), released from the keratinocytes which constitute the squamous epithelium.

On the basis of the information presented in the present study and based on a review of the literature, we propose a hypothesis for the mechanism of pathogenesis in nasal polyps: In the normal nasal mucosa, DCs are infrequently identified scouting for pathogens. When invasion by a pathogen occurs, different subsets of the DCs expressed various types of Toll-like receptors (TRLs) to combine with the pathogen-associated molecular patterns (PAMPs) and became mature themselves (9). In a previous study, Claeys *et al*(10) observed that TRL-2 and TRL-4 were expressed in the lamina propria below the mucous membrane of the nasal polyp tissue and that DCs presented the MHC II-antigenic epitope to T cells. The DCs were then connected with the T cells by the CD40/CD40L cell surface molecules. Next, the B7 molecules (CD80/CD86) on the surface of the DCs combined with the CD28/CTLA-4 of the T cells to induce the activation of the T cells, causing an immunoreaction. The result was that either the DCs or T cells became mature so that they were able to release chemokines to attract more DCs and T cells to assemble in the pathogenic tissues. This enhanced the cycle leading to increases in the quantities of DCs (as shown in the present study) and T cells (11) in the nasal polyps. The effects of the hyperplastic quantities of DCs and T cells then followed.

Firstly, hyperplastic quantities of DCs and T cells are the main cause of an imbalance in the proportion of Th1/Th2 cells (12) and the predominance of Th2 cell cytokines (13). This may involve the following mechanism: DCs are the main infection factors for the differentiation of Th0 (14). DCs are of two types; myeloid-derived DCs (DC1) and lymphoid-related DCs (DC2). In general, DC1 lead to the preferential development of a Th1 response and DC2 to a Th2 response (15). A study by Jahnsen *et al* identified that the amount of DC2 was increased in inflammatory and antigen-stimulated tissues. DC2 induced a Th2 response; the number of Th2 cells increased and the expression of Th2 cytokines was enhanced (16). Alternatively, different subsets of the DCs express varying types of TRLs to lead to different immunoreaction types. It has been reported that low lipopolysaccharide (LPS) levels led to a Th2 response by TRL-4 and that mice expressed a strong Th2 response following immunization with TRL-2 ligands and ovalbumin (OVA) (17). Nasal polyps express high levels of TRL-2 and TRL-4 (18) which are relevant to the Th2 response. The DCs induce the maturation and differentiation of T cells using cytokines and co-stimulating molecules on the cell surface. The CD80 of the DCs act as the co-stimulating molecule of the Th1 cells while CD86 belongs to the Th2 cells. In the nasal polyp tissues, DCs induce an imbalance in the proportion of the Th1/Th2 cells by causing the expression of CD80/CD86 to become unbalanced (19). The reciprocal interaction of the DCs and T cells through CD40/CD40L may induce DCs to secrete IL-12 (20). IL-12 is the most significant cytokine in the transformation of Th0 cells to Th1 cells. However, scholars have demonstrated that IL-10 is highly expressed in nasal polyp tissues (21). DCs are not only able to secrete IL-10 themselves but are also able to induce T cells to secrete IL-10 (15). IL-10 may restrain the Th1 cell cytokines. Reider *et al*(22) demonstrated that IL-10 could decrease the capacity of DCs in secreting IL-12 so that the differentiation from Th0 to Th1 was restrained but differentiation to Th2 was induced. Nasal polyps highly express TRL-2 (10,18) which in turn induces a Th2 immune response (17). DCs which express TRL-2 are able to induce T cells to secrete IL-4 (17). IL-4 is the most significant cytokine in the transformation of Th0 cells to Th2 cells. In nasal polyp tissues, IL-4 levels are increased (7) and cause the Th0 cells to transform into Th2 cells. Th2 is able to secrete IL-4 itself. This process became a cycle that encourages the predominance of the cytokines produced by the Th2 cells in the nasal polyps. Otherwise, IL-4 encourages the B cells to transform into plasmocytes which secrete IgE (23). The high level of IgE has been reported to be one of the demonstrably correlative factors in nasal polyp pathogenesis and has been identified to be positively correlated with EOS infiltration (6).

In the nasal polyp tissues, the reciprocal control of the DCs and T cells, through CD40/CD40L, B7-CD28/CTLA-4 co-stimulating molecules, the high levels of IL-4 and IL-10 and the low expresson levels of IL-12, induced an imbalance in the proportion of the Th1/Th2 cells and caused the predominance of Th2 cell cytokines.

Secondly, the hyperplastic quantities of the DCs and T cells cause high expression levels of IL-5 in the nasal polyp tissues; active DCs induce CD4+ T cells to secrete IL-5 (15,24). In previous studies, the high level of IL-5 has been identified to be one of the most significant demonstrably correlative factors with nasal polyps and one of the promotory factors in EOS infiltration (5,13). In the initial stages, IL-5 is generated mostly by Th2 cells. As the nasal polyps develop, EOSs replace the Th2 cells to become the most significant source of IL-5. Yi et al (25) demonstrated that IL-5 was able to restrain the maturation of DCs directly. In nasal polyp tissues, DCs induced T cells to secrete IL-5 by increasing the amount of EOS infiltration. The infiltrating EOSs secrete IL-5 to restrain the maturation of the DCs. This allows the DCs to pause in their juvenile stage, encouraging an effective immunoreaction and the development of nasal polyps.

In conclusion, the present study identified that there was a large quantity of DC infiltration in the nasal polyp tissues. The DCs recognized the PAMPs via TRLs and initiated the immunoreaction of the nasal polyps. Next, the DCs induced the infiltration and differentiation of T cells using co-stimulating molecules and cytokines. Following this, the DCs induced an imbalance in the proportion of the Th1/Th2 cells (12) and the predominance of Th2 cell cytokines through cell and serum mechanisms. In addition, the DCs were able to induce the infiltration of the EOSs by IL-5.

## Figures and Tables

**Table I:** S-100 expression in the nasal polyps and normal inferior turbinate groups.

Group	Positive rate (%)	Frowits’ method			IOD
		−	+	++	
Normal inferior turbinate (n=10)	10.0	9	1	0	890.02±723.24
Nasal polyps (n=45)	88.9	5	19	21	8540.12±1249.79
P-value			<0.01		<0.01

IOD, integrated optical density.

**Table II:** Area, number and density of the double stained dentritic cells (DCs).

Group	Positive rate (%)	Total area of double stained cells (cells/mm^2^)	Total number of double stained cells	Density of double stained cells (cells/mm^2^)
Normal inferior turbinate (n=10)	10.0	299.3±177.6	46.3±38.3	589±456
Nasal polyps (n=45)	88.9	3605.2±796.2	664.3±215.3	7125±2575
P-value		<0.01	<0.01	<0.01
